# Molecular Characterization of AMPA-Receptor-Containing Vesicles

**DOI:** 10.3389/fnmol.2021.754631

**Published:** 2021-10-15

**Authors:** John Jacob Peters, Jeremy Leitz, Juan A. Oses-Prieto, Alma L. Burlingame, Axel T. Brunger

**Affiliations:** ^1^Department of Molecular and Cellular Physiology, Stanford University, Stanford, CA, United States; ^2^Department of Neurology and Neurological Sciences, Stanford University, Stanford, CA, United States; ^3^Department of Structural Biology, Stanford University, Stanford, CA, United States; ^4^Department of Photon Science, Stanford University, Stanford, CA, United States; ^5^Howard Hughes Medical Institute, Stanford University, Stanford, CA, United States; ^6^Department of Pharmaceutical Chemistry, University of California, San Francisco, San Francisco, CA, United States

**Keywords:** AMPAR trafficking, synaptic plasiticity, proteomics, vesicle fusion, SNAREs

## Abstract

Regulated delivery of AMPA receptors (AMPARs) to the postsynaptic membrane is an essential step in synaptic strength modification, and in particular, long-term potentiation (LTP). While LTP has been extensively studied using electrophysiology and light microscopy, several questions regarding the molecular mechanisms of AMPAR delivery *via* trafficking vesicles remain outstanding, including the gross molecular make up of AMPAR trafficking organelles and identification and location of calcium sensors required for SNARE complex-dependent membrane fusion of such trafficking vesicles with the plasma membrane. Here, we isolated AMPA-containing vesicles (ACVs) from whole mouse brains *via* immunoisolation and characterized them using immunoelectron microscopy, immunoblotting, and liquid chromatography–tandem mass spectrometry (LC–MS/MS). We identified several proteins on ACVs that were previously found to play a role in AMPAR trafficking, including synaptobrevin-2, Rabs, the SM protein Munc18-1, the calcium-sensor synaptotagmin-1, as well as several new candidates, including synaptophysin and synaptogyrin on ACV membranes. Additionally, we identified two populations of ACVs based on size and molecular composition: small-diameter, synaptobrevin-2- and GluA1-containing ACVs, and larger transferrin- receptor-, GluA1-, GluA2-, and GluA3-containing ACVs. The small-diameter population of ACVs may represent a fusion-capable population of vesicles due to the presence of synaptobrevin-2. Because the fusion of ACVs may be a requisite of LTP, this population could represent trafficking vesicles related to LTP.

## Introduction

At glutamatergic synapses, AMPA receptors (AMPARs) are responsible for the largest component of postsynaptic responses in the form of cation influx, and along with NMDARs, are major contributors to various forms of synaptic plasticity including long-term potentiation (LTP) (Dingledine et al., 1999; Malinow and Malenka, 2002; Bredt and Nicoll, 2003; Collingridge et al., 2004; Shepherd and Huganir, 2007; Newpher and Ehlers, 2008). Upon the arrival of an action potential, glutamate is released from synaptic vesicles into the synaptic cleft where it binds to postsynaptic AMPARs. When bound with glutamate, AMPARs open, allowing cations to enter and depolarize the postsynaptic cell. As a requisite of LTP (Malinow and Malenka, 2002), the cellular correlate of memory (Nabavi et al., 2014), AMPAR trafficking vesicles (ATVs) are exocytosed and AMPARs are recruited to the synapse, increasing the postsynaptic response (Lledo et al., 1998). The increased presence of AMPARs in the postsynaptic membrane has been characterized by light microscopy and electrophysiology studies, but little is known about the molecular composition of ATVs and the process by which they exocytose at the plasma membrane (Noel et al., 1999; Shi et al., 1999; Takumi et al., 1999; Liu and Cull-Candy, 2000; Passafaro et al., 2001; Ju et al., 2004). AMPA receptors at the synapse come from two sources: receptors that have been recycled from the plasma membrane and receptors that have been synthesized *de novo*. Regardless of etiology, AMPARs are trafficked in ATVs before they are inserted into the plasma membrane in a SNARE-dependent process (Jurado et al., 2013; Wu et al., 2017). While much is known about SNARE-dependent membrane fusion elsewhere in neurons (e.g., during neurotransmitter release *via* synaptic vesicle exocytosis), AMPAR insertion *via* ATV fusion has only recently begun to be elucidated. The insertion of AMPARs during LTP is particularly intriguing due to evidence that the process is calcium-triggered and involves synaptotagmins (Wu et al., 2017). Electrophysiology studies revealed that syntaxin 3 (Stx-3), SNAP-47, and synaptobrevin 2 (Syb2) are SNARE proteins involved in ATV fusion during LTP and that synaptotagmin-1 (Syt1) and −7 (Syt7) are the calcium sensors for this process (Jurado et al., 2013; Wu et al., 2017). Rab proteins, including Rab5, Rab8, Rab11, and Rab39, and the transferrin receptor (TfR) also play a key role in AMPAR delivery to synapses (Gerges et al., 2004; Liu et al., 2016). Despite these discoveries, there are many outstanding questions surrounding the ATV lifecycle, from ATV fusion to AMPAR endocytosis. For example, the cellular localization of most synaptotagmins is unknown. While Syt1, a key synaptotagmin involved in synaptic vesicle fusion, and other synaptotagmins have been found on synaptic vesicles, it is not known whether synaptotagmins are likewise trafficked on ATVs. Moreover, it is unclear to what extent proteins are sorted as AMPARs are endocytosed, stored in recycling endosomes, and inserted back into the postsynaptic membrane.

Due to their small size, relatively low abundance (compared to synaptic vesicles), and relative transience *in vivo*, ATVs have been challenging to study (Kittler and Moss(eds), 2006). Electron microscopy studies have yet to uncover convincing evidence of ATVs at the synapse perhaps because deliveries of AMPARs to the postsynaptic membrane often happen after induction of synaptic plasticity. The transience of AMPAR delivery and the difficulty of specifically targeting synapses that are undergoing plasticity with electron microscopy makes studying the molecular components involved in AMPAR trafficking *in situ* challenging. Advances in organelle isolation from synaptosomes have made it possible to faithfully isolate small organelles, specifically synaptic vesicles, for molecular characterization (Ahmed et al., 2013). To overcome the problems associated with studying AMPAR trafficking *in vivo*, we have adopted a similar strategy to specifically isolate AMPA-containing vesicles (ACVs) from synaptosomes purified from whole mouse brains. Subcellular fractions were purified from neurons using multiple rounds of differential centrifugation, after which AMPAR-containing components were immunoprecipitated with a GluA1 antibody and then isolated by specific elution with a peptide that competes with the GluA1 subunit of AMPARs. The resulting sample was characterized using immunoblotting, liquid chromatography–tandem mass spectrometry (LC–MS/MS), and immunoelectron microscopy. Here, we offer the first unbiased characterization of GluA1-containing ACVs. LC–MS/MS confirms several previously identified proteins found to be involved in AMPAR trafficking and identifies potential new candidates for AMPAR receptor trafficking. Immunoelectron microscopy reveals heterogenous populations of ACVs in terms of protein compositions and vesicle diameters. Combined, these data offer an unbiased candidate list of proteins potentially involved in AMPAR receptor trafficking.

## Materials and Methods

### Animal Ethics Statement

The animal study was reviewed and approved by the Administrative Panel on Laboratory Animal Care (APLAC) at Stanford University (IACUC #29981).

### Purification of AMPA-Containing Vesicles

**FIGURE 1:**
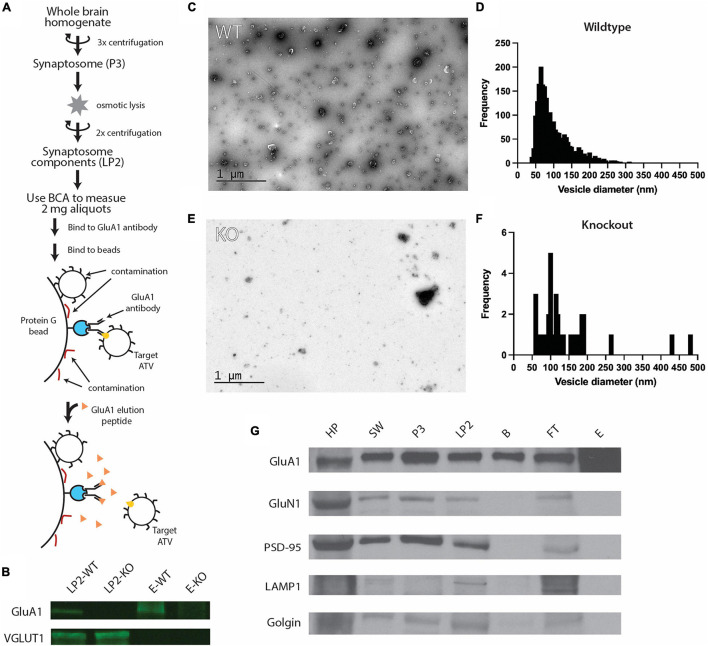
Purification of ACVs from whole mouse brain. **(A)** Purification protocol for isolating ACVs. At variance to all previous methods, the final step of the preparation involves elution with a GluA1 peptide that corresponds to the epitope of the GluA1 monoclonal antibody. Note that the same amount of protein (as assessed by BCA) was inputted into the same immunoisolation step for both wild-type and knockout preparations. **(B)** Western blots for GluA1 and VGLUT1 in WT isolated synaptosome content (LP2-WT), GluA1 KO isolated synaptosome content (LP2-KO), GluA1 peptide eluate from wild-type mice (E-WT), and GluA1 peptide eluate from GluA1 KO mice (E-KO). Original blots provided in Supplementary Figure 1. **(C)** Negative stain electron microscopy image of GluA1 peptide eluate of wild-type mice. Additional images are shown in Supplementary Figure 2. All images are provided as Source Data. **(D)** Histogram of vesicle diameters from wild-type eluate from three independent immunoisolations. **(E)** Negative stain electron microscopy image of GluA1 peptide eluate of GluA1 KO mice. Additional images are shown in Supplementary Figure 3. Additional images are provided in Supplementary Material. **(F)** Histogram of vesicle diameters from knockout eluate from three independent immunoisolations. **(G)** Representative western blots from three independent immunoisolations for GluA1 (102 kD), GluN1 (115 kD), PSD-95 (98 kD), LAMP1 (130 kD), and Golgin (100 kD) for homogenized whole brain pellet (HP), the second synaptosome wash step (SW), synaptosome (P3), synaptosome content (LP2), beads from immunoisolation prior to elution **(B)**, flowthrough from immunoisolation (FT), and GluA1-peptide eluate off beads **(E)** for wild-type mice. Original blots provided in Supplementary Figure 4.

To isolate ACVs, we followed a previously developed protocol for synaptosome generation and synaptic vesicle isolation (Ahmed et al., 2013) and extensively modified it to specifically purify ACVs. Eight to twelve ∼P20 CD-1 mice were anesthetized using isoflurane in an open-drop chamber, and whole brains were immediately removed and homogenized. (See Figure 1 for full summary). This initial homogenate was spun in a JA-20 rotor at 2700 RPM (880 G) for 10 min to pellet blood vessels and other large cellular debris. The supernatant was then spun at 10,000 RPM (12,064 G) for 15 min to pellet synaptosomes. The supernatant was discarded and the periphery of the pellet was resuspended, which helps to remove mitochondria, before spinning at 11,000 RPM (14,597 G) for 15 min. The supernatant was again discarded, and the pellet resuspended to 5 ml total volume. The suspension was added to a Dounce homogenizer along with 45 ml of ultrapure water and was briefly homogenized to hypoosmotically lyse the synaptosomes. Immediately afterward, 60 μl of 1 mg/ml pepstatin A and 120 μl of 200 mM PMSF in 1 M HEPES was added. This solution was spun at 19,500 RPM (45,871 G) for 20 min to pellet plasma membrane and large cellular debris while leaving small organelles like vesicles in solution (LP1 for “lysis pellet 1”). The supernatant was then removed and spun in a Ti-70 ultracentrifuge at 50,000 RPM (256,631 G) for 2 h at 4°C to pellet small organelles like trafficking vesicles (LP2 for “lysis pellet 2”). The LP2 pellet was transferred to a small homogenizer and resuspended in 2 ml of PBS by homogenization and mechanically sheared through a 27-gauge needle. The concentration of LP2 was determined using BCA and aliquoted into 2 mg aliquots at approximately 5 μg/μl. Any LP2 not used immediately for ACV isolation was flash frozen with liquid nitrogen and stored at −80°C until use.

To isolate ACVs from LP2, 1 aliquot of 2 mg LP2 was diluted to 1 ml total volume in 0.5% BSA in PBS, 5 μl of mouse anti-GluA1 monoclonal antibody (1 μg/μl, Synaptic Systems, Gottingen, Germany) was added and allowed to bind while rotating for 12 h at 4°C. To prevent non-specific binding, 50 μl of paramagnetic protein G beads (Dynabeads, ThermoFisher Scientific, Waltham, MA, United States) were washed three times in 0.5% BSA in PBS for 15 min on ice and then three times in PBS for 5-min washes on ice prior to addition of LP2. The LP2 mixture was then added to the beads and rotated for 2 h at 4°C. Dynabeads were separated from solution using a magnet, and the flow through was collected for western blot analysis. ACVs were then gently eluted with three, 20-min washes with 33 μl of GluA1 peptide (20 μg/μl) representing the same synthetic peptide the antibody was created against (sequence: SHSSGMPLGATGL) (GenScript Biotech, Piscataway, NJ, United States). ACVs were then immediately used and continually stored on ice at 4°C. Protein concentration was measured by Bradford assay. Serial dilutions of BSA were used to generate a standard curve.

### GluA1 Knockout Mice

Knockout mutant mice for *GRIA1*, the gene encoding GluA1, have been previously described (Zamanillo et al., 1999). Knockout mice were generated by interbreeding heterozygous mice. The same immunoisolation protocol was used as for wild-type mice.

### Western Blots

For western blot analysis, samples were first separated by SDS-PAGE and then electrophoretically transferred onto membranes. After transfer, the membranes were then treated with blocking buffer and labeled using an iBind Flex (ThermoFisher Scientific). GluA1 (Abcam – ab1504, rabbit, 1:2,000, Cambridge, United Kingdom), GluN1 (Synaptic Systems – 114-003, rabbit, 1:1,000), PSD-95 (Abcam – ab18258, rabbit, 1:2,000), VGLUT1 (Abcam – ab77822, rabbit, 1:1,000), Lamp1 (Proteintech – 21997-1-AP, rabbit, 1:2,000, Rosemont, IL, United States), and golgin (Abcam – 84380, rabbit, 1:2,000) were each individually probed. A goat-anti rabbit secondary antibody conjugated with HRP was used for all chemiluminescent western blots (Abcam – ab672, 1:50,000), and a goat-anti rabbit secondary antibody conjugated with IRDye 800CW was used for all fluorescent western blots (Abcam – ab216773, 1:50,000. The bands were visualized either by immunofluorescence with a LI-COR Odyssey (Lincoln, NE, United States) or with chemiluminescence with a Konica Minolta – SRX101A (Tokyo, Japan). All antibodies were diluted from 1 mg/ml stock.

### Transmission Electron Microscopy

Negative stain transmission electron microscopy (TEM) was performed on ACVs. Copper mesh grids were glow discharged in argon gas for 20 s before 4 μl of ACV eluate was applied and allowed to settle for 30 min. The grid was then washed three times with ultra-pure water. The grid was negatively stained using 1% uranyl acetate for 2 min then blotted and allowed to dry at room temperature for 20 min. The grid was imaged using a JEOL 1400 TEM at 120 keV. The diameters of ACVs were measured using ImageJ. Two diameters were measured using the line segment tool in ImageJ for each ACV; each measurement was scaled using the scale bar as reference for each given image. The two diameters were averaged together to get a final diameter. Immunogold labeling was performed for GluA2 (BioLegend, San Diego, CA, United States), GluA3 (Synaptic Systems), Syb2 (Abcam), Syt1 (Abcam), TfR (ThermoFisher Scientific), and Syp1 (Synaptic Systems). For immunogold labeling, the same protocol for negative stained TEM was performed; however, after ACV addition, the grids were incubated in a 1:50 dilution of rabbit polyclonal primary antibody in blocking buffer (0.5% BSA, 0.5% ovalbumin in PBS) for 1 h. Then three, 5-min washes in PBST were performed followed by a 1-h incubation in 1:50 10 nm gold goat anti-rabbit secondary antibody (Electron Microscopy Sciences – 25108, Hatfield, PA, United States). Three more 5-min washes in PBST were performed, and then samples were fixed in 8% glutaraldehyde for 30 s. Staining and imaging were performed as previously described.

### Liquid Chromatography–Mass Spectrometry

Purified ACVs were resuspended in 50 μl 0.2% Rapigest (Waters, Milford, MA, United States) in 20 mM NH4HCO3 in 0.65 ml low protein binding polypropylene tubes before the addition of 5 mM DTT and incubation at 60°C for 30 min. After this, iodoacetamide was added to a final concentration of 7.5 mM and samples were incubated for 30 additional minutes. Samples were then digested with 2.5 μg of sequencing grade trypsin (Trypsin Gold, Mass spectrometry grade, Promega, Madison, WI, United States) at 37°C, overnight. A second aliquot of trypsin (1.5 μg) was added, and the samples incubated for an additional 3 h at 37°C. After this, samples were acidified by adding 5% formic acid and incubated for 30 min at room temperature. Tryptic peptides were recovered from the supernatant by C18 solid phase extraction using ZipTips (MilliporeSigma, Burlington, MA, United States), eluted in two, 7 μl drops of 50% acetonitrile and 0.1% formic acid, and evaporated and resuspended in 5 μl 0.1% formic acid for LC–MS/MS analysis.

Peptides resulting from trypsinization were analyzed on a QExactive Plus mass spectrometer (ThermoFisher Scientific) connected to a NanoAcquity Ultra Performance UPLC system (Waters). A 15-cm EasySpray C18 column (ThermoFisher Scientific) was used to resolve peptides (60-min 2–30% B gradient with 0.1% formic acid in water as mobile phase A and 0.1% formic acid in acetonitrile as mobile phase B, at a flow rate of 300 nl/min). MS was operated in data-dependent mode to automatically switch between MS and MS/MS. MS spectra were acquired between 350 and 1,500 m/z with a resolution of 70,000. For each MS spectrum, the top 10 precursor ions with a charge state of 2+ or higher were fragmented by higher-energy collision dissociation. A dynamic exclusion window was applied which prevented the same m/z from being selected for 10 s after its acquisition.

Peak lists were generated using PAVA in-house software (Guan et al., 2011). All generated peak lists were searched against the mouse subset of the UniProtKB database (SwissProt.2013.6.17) (plus the corresponding randomized sequences to calculate false discovery rate on the searches), using Protein Prospector (Clauser et al., 1999). The database search was performed with the following parameters: a mass tolerance of 20 ppm for precursor masses and 30 ppm for MS/MS, cysteine carbamidomethylation as a fixed modification, and acetylation of the N terminus of the protein, pyroglutamate formation from N terminal glutamine, and oxidation of methionine as variable modifications. A 1% false discovery rate was permitted at the protein and peptide level. All spectra identified as matches to peptides of a given protein were reported, and the number of spectra (peptide spectral matches, PSMs) was used for label free quantitation of protein abundance in the samples. Abundance index for each protein was calculated as the ratio of PSMs for a protein to the total PSMs for all components identified in the run divided by the polypeptide molecular weight.

### Additional Statistics

The Kolmogorov–Smirnov test was performed to test statistical significance between an independent population of vesicles from the gross population of all ACVs isolated (Figure 1D, from three independent immunoisolations) and vesicles positively labeled with gold-conjugated antibodies against Syb2 (*p* = 0.0101, three immunoisolations), Syt1 (*p* = 0.9382, three immunoisolations), Syp1 (*p* < 0.0001, four immunoisolations), TfR (*p* = 0.0100, two immunoisolations), GluA2 (*p* < 0.0001, three immunoisolations), and GluA3 (*p* < 0.0001 three immunoisolations).

## Results

### AMPA-Containing Vesicle Isolation From Whole Mouse Brains

To characterize the molecular composition of ACVs, synaptosomes were purified from whole brains of 6–12 P20 mice and hypoosmotically lysed to release their contents (Ahmed et al., 2013). The resulting lysis pellet (LP2), comprised of synaptosome contents, was flash frozen and stored at −80°C until used. GluA1-containing components were first extracted from LP2 using an anti-GluA1 antibody (Figure 1A). Antibody was allowed to bind overnight at 4°C and was subsequently bound to protein G paramagnetic beads before ACVs were gently eluted by competing with a peptide that contains the GluA1 antibody epitope to allow for specific elution and isolation. As such, this elution is based on competition between GluA1 and the peptide which is present in large molar excess. Thus, contaminants that do not specifically bind to the antibody recognition site, should remain on the beads. Western blot analysis confirmed the presence of GluA1 in LP2 and the eluate (Figure 1B and Supplementary Figure 1). Additionally, western blot analysis confirmed the presence of VGLUT1, a marker of glutamatergic synaptic vesicles (a potential contaminate), in LP2 but not in the eluate. Negative stain electron micrographs (Figure 1C and Supplementary Figure 2) revealed that the purification yielded vesicles with a diameter of 102.7 ± 50.8 nm (arithmetic mean) (Figure 1D), marking the first time ACVs (including ATVs) have been visualized. To further confirm the fidelity of the ACV preparation, the same immunoprecipitation and GluA1 peptide elution protocol was performed using LP2 purified from *GLUA1−/−* knockout mice. Western blot analysis confirmed the deletion of *GLUA1* but the retention of VGLUT1 expression (Figure 1B). There were substantially fewer vesicles identified in the sample isolated from knockout animals as assessed by negative stain electron microscopy (Figure 1E and Supplementary Figures 2, 3): Immunoisolation from wild-type mice yielded 4.83 vesicles/μm^2^ (5 micrographs, 535 vesicles in 110.8 μm^2^), while immunoisolation from knockout mice yielded 0.30 vesicles/μm^2^ (5 micrographs, 34 vesicles in 112.0 μm^2^) (for all images, see Source Data); note that the same amount of protein (as assessed by BCA) was inputted into the same immunoisolation step for both wild-type and knockout preparations. Additionally, we measured the total protein concentration in the elution by Bradford assay and found the wild-type eluate contained ∼35.3 μg/ml compared to GLUA1*−*/*−* knockout eluate which contained only ∼5.8 μg/ml. It is important to note that due to the size and amino acid composition of the elution peptide, the elution peptide itself does not provide a detectable signal in the Bradford assay. For both wild-type and knockout preparations, defined aliquots of 2 mg of total protein LP2 were inputted into the same immunoisolation procedure, so the decreased yield from immunoisolation from the knockout LP2 is indicative of a decreased amount of GluA1-containing material. Therefore, our immunoisolation procedure targets ACVs (including ATVs) and minimizes contamination by other components.

### Immunoisolation Leads to Pure AMPA-Containing Vesicles

While initial results were suggestive of a relatively pure population of ACVs, we probed several additional molecules to further confirm eluate quality. Western blots were performed on samples from each step of the isolation process to monitor which molecular components were enriched (Figure 1G). Confirming previous results, the GluA1 subunit of the AMPAR was identified throughout the isolation process and was enriched in the final eluate. Several other proteins were probed to verify isolation purity, including GluN1, PSD-95, LAMP1, and golgin. GluN1 is an NMDA receptor subunit and is also present in the glutamatergic postsynaptic compartment (Paoletti et al., 2013). Similarly, PSD-95 is a component of the postsynaptic density at excitatory synapses (Craven and Bredt, 1998). LAMP1 is a lysosomal marker (Griffiths et al., 1988), and golgin is a Golgi apparatus marker (Munro, 2011). All these markers were identified in each step until the elution step with GluA1 peptide, indicating that as expected, subcellular compartments, including postsynaptic plasma membrane components, were maintained throughout the preparation but were excluded upon the specific GluA1 peptide elution step.

### Immunoelectron Microscopy Revealed Molecular Components of AMPA-Containing Vesicles

**FIGURE 2:**
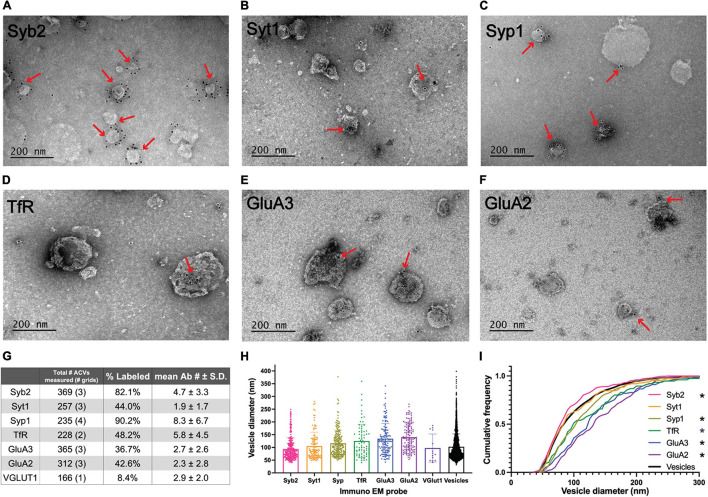
Electron microscopy analysis of ACV samples. **(A–F)** Immuno-negative stain electron micrographs for GluA2, GluA3, Syb2, Syt1, TfR, and Syp1. Red arrows indicate regions with gold-conjugated secondary antibody. **(G)** Summary table of negative stain electron microscopy analysis of antibody labeled preparations of GluA1 peptide eluate of wild-type mice. Total number of ACVs represents all ACVs measured for the given probe. Each grid represents an independent preparation and imaging experiment (all images are provided as Source Data). **(H)** Mean and standard deviations of diameters of vesicles labeled with each antibody. **(I)** Normalized cumulative frequency distributions of diameters of vesicles labeled with each antibody. The bold line represents the frequency distribution of all vesicles from Figure 1D. The Kolmogorov–Smirnov test was performed to test statistical significance between an independent population of vesicles from Figure 1D and vesicles containing Syb2 (*p* = 0.0101), Syt1 (*p* = 0.9382), Syp1 (*p* < 0.0001), TfR (*p* = 0.0100), GluA2 (*p* < 0.0001), and GluA3 (*p* < 0.0001). *Indicates *p*-value < 0.05.

**FIGURE 3:**
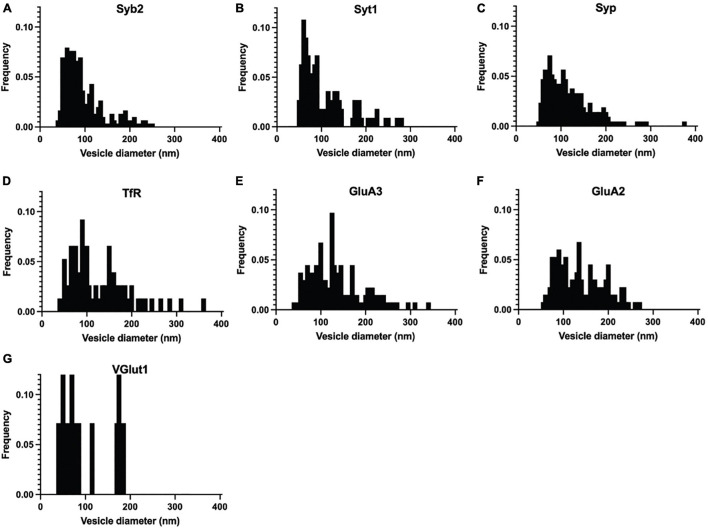
Normalized frequency distribution of diameters of immuno-labeled ACVs identified in negative stain electron microscopy images of GluA1-peptide eluate from wild-type mice. **(A)** Normalized frequency distribution of vesicles positive for Syb2 (303 vesicles from 3 independent ACV purifications). **(B)** Syt1 (113 vesicles from 3 independent ACV purifications) **(C)** Syp1 (212 vesicles from 4 independent ACV purifications) **(D)** TfR (110 vesicles from 2 independent ACV purifications) **(E)** GluA3 (134 vesicles from 3 independent ACV purifications) **(F)** GluA2 (133 vesicles from 3 independent ACV purifications) **(G)** VGlut1 (14 vesicles from 1 independent ACV purifications).

Immunoelectron microscopy was performed on the isolated ACVs to assess the frequency of protein localization on ACVs for several known AMPAR-associated proteins (Figures 2, 3). Secondary antibody concentration was optimized to minimize non-specific, background gold (<1 free gold per field of view). A positive hit was defined as a gold particle within 5 nm of an ACV. AMPAR subunits GluA2 and GluA3 were probed to test for the presence of these subunits in the GluA1-affinity purified ACVs. GluA2 was found on 42.6% of ACVs, and the GluA3 subunit was found on 36.7% of ACVs. TfR, a known marker of AMPAR endosomes, was identified on 48.2% of ACVs. Synaptophysin 1 (Syp1) was identified on 90.2% of vesicles. Syb2 was found 82.1% of ACVs, while Syt1 was identified on 44.0% of ACVs. Additionally, the diameters of ACVs that were labeled by GluA2 (140.1 ± 52.5 nm), GluA3 (134.5 ± 60.2 nm), TfR (121.7 ± 66.4 nm), Syp1 (116.2 ± 50.8 nm), Syb2 (93.9 ± 43.8 nm), and Syt1 (105.2 ± 54.6 nm) were measured (all arithmetic means) (Figure 2H). As a negative control, VGLUT1 (vesicular glutamate transporter), a marker of glutamatergic synaptic vesicles, was probed (data not shown), and only 8.4% of ACVs were positive for VGLUT1. The Kolmogorov–Smirnov test was performed, comparing the cumulative frequency distribution for each marker to the overall population of ACVs obtained from the negative stain experiments shown in Figure 1D (Figure 2I). The cumulative frequency distribution for Syb2-labeled ACVs was significantly shifted to the left, indicating smaller diameters (*p* = 0.0101), while the Syp1 (*p* < 0.0001), TfR (*p* = 0.0100), GluA2 (*p* < 0.0001), and GluA3 (*p* < 0.0001) distributions were significantly shifted to the right (larger diameters). Syt1 was not significantly shifted from the global ACV diameter distribution (*p* = 0.9382). Smaller, Syb2-labeled vesicles are unlikely to be synaptic vesicles due to the low frequency of VGLUT1-labeled vesicles and the substantial difference in size between Syb2-labeled vesicles and the 40-45 nm diameter that has previously been reported for synaptic vesicles (Takamori et al., 2006). Additionally, the mean diameter of VGLUT-1 labeled vesicles (arithmetic mean of 97.6 ± 54.8 nm) is also much larger than the reported diameters of synaptic vesicles, which suggests that the small population of VGLUT1-labeled vesicles are most likely small endosomes or membrane fragments.

### Liquid Chromatography-Tandem Mass Spectrometry Analysis Identifies Known AMAP Receptor Trafficking Proteins and Candidates for New Proteins

**FIGURE 4:**
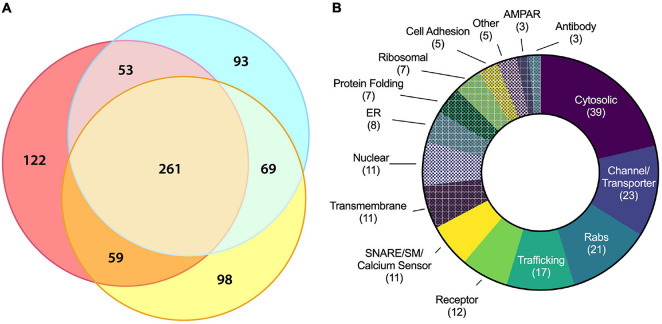
Molecular characterization of ACV proteins using LC–MS/MS. **(A)** Three-way Venn diagram showing protein hits in three LC–MS/MS biological replicates with each color representing a biological replicate. **(B)** Protein ontology of the 180 identified candidates using gene ontology resource. See Table 1 for all data.

Liquid chromatography–tandem mass spectrometry was performed on isolated ACVs. We identified a total of 755 unique proteins with expectation values <0.005 across three biological replicates (Fenyö and Beavis, 2003). We applied two additional filters to these 755 proteins to ensure high quality and abundance. Of those 755 unique proteins, 442 proteins were identified in two or more data sets (Figure 4A). The sequence coverage (fraction of protein sequence that was identified) for 180 proteins was greater than 7.5%, suggestive of higher abundance. Proteins were manually categorized based on function and cellular localization (Figure 4B). Cytosolic proteins, channels/transporters, and Rabs were the most commonly identified protein classes with 39, 23, and 21 hits, respectively. Among the top proteins enriched in ACVs (Table 1) are AMPAR subunits GluA1, GluA2, and GluA3, as well as AMPAR-associated Dnajc13 (Perrett et al., 2015), TfR (Liu et al., 2016), neuroplastin (Jiang et al., 2021), and ABHD6 (Wei et al., 2016). In addition, the genes for Rab5, 8, 11, and 39, all implicated in AMPAR trafficking, were also among the top 180 candidates (Gerges et al., 2004). Furthermore, other synaptic proteins that have yet to be identified as AMPAR-trafficking-associated, including Syp1, synaptogyrin-1 (Syngr1), and −3 (Syngr3), and Munc18-1, were identified (Table 1).

**TABLE 1:** Protein ontology of proteins in ACVs identified with LC–MS/MS using gene ontology resource.

Gene name	Protein name
**AMPAR subunit**	
GRIA1	Glutamate receptor, ionotropic, AMPA1 (alpha 1)
GRIA2	Glutamate receptor, ionotropic, AMPA2 (alpha 2)
GRIA4	Glutamate receptor, ionotropic, AMPA4 (alpha 4)
**Calcium sensor**	
SYT1	Synaptotagmin I
SYT2	Synaptotagmin II
**Cell adhesion**	
BSG	Basigin
NCAM1	Neural cell adhesion molecule 1
NEGR1	Neuronal growth regulator 1
NPTN	Neuroplastin
THY1	Thymus cell antigen 1, theta
**Channel/transporter**	
ATP1A1	ATPase, Na+/K+ transporting, alpha 1 polypeptide
ATP1A2	ATPase, Na+/K+ transporting, alpha 2 polypeptide
ATP1A3	ATPase, Na+/K+ transporting, alpha 3 polypeptide
ATP1B1	ATPase, Na+/K+ transporting, beta 1 polypeptide
ATP1B2	ATPase, Na+/K+ transporting, beta 2 polypeptide
ATP1B3	ATPase, Na+/K+ transporting, beta 3 polypeptide
ATP2A2	ATPase, Ca++ transporting, cardiac muscle, slow twitch 2
ATP2B1	ATPase, Ca++ transporting, plasma membrane 1
ATP2B2	ATPase, Ca++ transporting, plasma membrane 2
ATP2B3	ATPase, Ca++ transporting, plasma membrane 3
ATP2B4	ATPase, Ca++ transporting, plasma membrane 4
ATP6V0A1	ATPase, H+ transporting, lysosomal V0 subunit A1
ATP6V0D1	ATPase, H+ transporting, lysosomal V0 subunit D1
ATP6V1A	ATPase, H+ transporting, lysosomal V1 subunit A
ATP6V1B2	ATPase, H+ transporting, lysosomal V1 subunit B2
ATP8A1	ATPase, aminophospholipid transporter (APLT), class I, type 8A, member 1
SLC12A5	Solute carrier family 12, member 5
SLC17A6	Solute carrier family 17 (sodium-dependent inorganic phosphate cotransporter), member 6
SLC17A7	Solute carrier family 17 (sodium-dependent inorganic phosphate cotransporter), member 7
SLC32A1	Solute carrier family 32 (GABA vesicular transporter), member 1
SLC6A17	Solute carrier family 6 (neurotransmitter transporter), member 17
VDAC1	Voltage-dependent anion channel 1
VDAC3	Voltage-dependent anion channel 3
**Cytosolic**	
ABHD6	Abhydrolase domain containing 6
ACSL6	Acyl-CoA synthetase long-chain family member 6
ACTB	Actin, beta
ADRBK2	G protein-coupled receptor kinase 3
AK5	Adenylate kinase 5
ALG2	Asparagine-linked glycosylation 2 (alpha-1,3-mannosyltransferase)
AP2A1	Adaptor-related protein complex 2, alpha 1 subunit
AP2A2	Adaptor-related protein complex 2, alpha 2 subunit
AP2M1	Adaptor-related protein complex 2, mu 1 subunit
APOE	Apolipoprotein E
ARF6	ADP-ribosylation factor 6
CALM1	Calmodulin 1
CAMK2A	Calcium/calmodulin-dependent protein kinase II alpha
CAMK2B	Calcium/calmodulin-dependent protein kinase II, beta
CAMK2G	Calcium/calmodulin-dependent protein kinase II gamma
CLTC	Clathrin, heavy polypeptide (Hc)
CNP	2′,3′-cyclic nucleotide 3′ phosphodiesterase
CYB5R3	Cytochrome b5 reductase 3
DAD1	Defender against cell death 1
DNM1	Dynamin 1
GAPDH	Glyceraldehyde-3-phosphate dehydrogenase
GDE1	Glycerophosphodiester phosphodiesterase 1
GDPD1	Glycerophosphodiester phosphodiesterase domain containing 1
HMOX2	Heme oxygenase 2
INA	Internexin neuronal intermediate filament protein, alpha
NCEH1	Neutral cholesterol ester hydrolase 1
NSF	*N*-ethylmaleimide sensitive fusion protein
PFKM	Phosphofructokinase, muscle
POR	P450 (cytochrome) oxidoreductase
PRKCG	Protein kinase C, gamma
PTPLAD1	3-Hydroxyacyl-CoA dehydratase 3
TUBA1A	Tubulin, alpha 1A
TUBA4A	Tubulin, alpha 4A
TUBB2A	Tubulin, beta 2A class IIA
TUBB4A	Tubulin, beta 4A class IVA
TUBB4B	Tubulin, beta 4B class IVB
TUBB5	Tubulin, beta 5 class I
UBB	Ubiquitin B
YWHAZ	Tyrosine 3-monooxygenase/tryptophan 5-monooxygenase activation protein, zeta polypeptide
**Endoplasmic reticulum**	
ATL1	Atlastin GTPase 1
CDIPT	CDP-diacylglycerol–inositol 3-phosphatidyltransferase (phosphatidylinositol synthase)
EMC9	ER membrane protein complex subunit 9
ERGIC1	Endoplasmic reticulum-golgi intermediate compartment (ERGIC) 1
ERLIN2	ER lipid raft associated 2
RCN2	Reticulocalbin 2
RPN1	Ribophorin I
TMEM33	Transmembrane protein 33
**Nuclear**	
CCAR1	Cell division cycle and apoptosis regulator 1
EMD	Emerin
ENDOD1	Endonuclease domain containing 1
H2AFV	H2A.Z histone variant 2
H2AFZ	H2A.Z variant histone 1
HIST1H2AB	H2A clustered histone 4
HIST1H2BF	H2B clustered histone 7
HIST1H4A	H4 clustered histone 1
HNRNPM	Heterogeneous nuclear ribonucleoprotein M
SFPQ	Splicing factor proline/glutamine rich (polypyrimidine tract binding protein associated)
TRP53I11	Transformation related protein 53 inducible protein 11
**Other**	
PLP1	Proteolipid protein (myelin) 1
PRSS1	Protease, serine 1 (trypsin 1)
SRSF3	Serine and arginine-rich splicing factor 3
TARDBP	TAR DNA binding protein
U2AF1	U2 small nuclear ribonucleoprotein auxiliary factor (U2AF) 1
**Protein folding**	
CANX	Calnexin
HSP90B1	Heat shock protein 90, beta (Grp94), member 1
HSPA5	Heat shock protein 5
HSPA8	Heat shock protein 8
PDIA3	Protein disulfide isomerase associated 3
TMX2	Thioredoxin-related transmembrane protein 2
VMA21	VMA21 vacuolar H+-ATPase homolog (*S. cerevisiae*)
**Rabs**	
RAB1	Ribonuclease, RNase A family 4
RAB10	RAB10, member RAS oncogene family
RAB11B	RAB11B, member RAS oncogene family
RAB13	RAB13, member RAS oncogene family
RAB14	RAB14, member RAS oncogene family
RAB15	RAB15, member RAS oncogene family
RAB18	RAB18, member RAS oncogene family
RAB1A	RAB1A, member RAS oncogene family
RAB1B	RAB1B, member RAS oncogene family
RAB2A	RAB2A, member RAS oncogene family
RAB35	RAB35, member RAS oncogene family
RAB39B	RAB39B, member RAS oncogene family
RAB3A	RAB3A, member RAS oncogene family
RAB3B	RAB3B, member RAS oncogene family
RAB3C	RAB3C, member RAS oncogene family
RAB5A	RAB5A, member RAS oncogene family
RAB6A	RAB6A, member RAS oncogene family
RAB6B	RAB6B, member RAS oncogene family
RAB7A	RAB7A, member RAS oncogene family
RAB8A	RAB8A, member RAS oncogene family
RAB8B	RAB8B, member RAS oncogene family
**Receptor**	
GNAI1	Guanine nucleotide binding protein (G protein), alpha inhibiting 1
GNAI2	Guanine nucleotide binding protein (G protein), alpha inhibiting 2
GNAO1	Guanine nucleotide binding protein, alpha O
GNAQ	Guanine nucleotide binding protein, alpha q polypeptide
GNB1	Guanine nucleotide binding protein (G protein), beta 1
GNB2	Guanine nucleotide binding protein (G protein), beta 2
LRP1	Low density lipoprotein receptor-related protein 1
M6PR	Mannose-6-phosphate receptor, cation dependent
P2RY12	Purinergic receptor P2Y, G-protein coupled 12
PGRMC1	Progesterone receptor membrane component 1
SORT1	Sortilin 1
TFRC	Transferrin receptor
**Ribosomal**	
EEF1A1	Eukaryotic translation elongation factor 1 alpha 1
RPL18	Ribosomal protein L18
RPL35A	Ribosomal protein L35A
RPL4	Ribosomal protein L4
RPL6	Ribosomal protein L6
RPL7	Ribosomal protein L7
RPLP0	Ribosomal protein, large, P0
**SNARE/SM**	
SNAP25	Synaptosomal-associated protein 25
STX12	Syntaxin 12
STX1A	Syntaxin 1A
STX1B	Syntaxin 1B
STX6	Syntaxin 6
STX7	Syntaxin 7
STXBP1	Syntaxin binding protein 1 (Munc18)
VAMP1	Vesicle-associated membrane protein 1
VAMP2	Vesicle-associated membrane protein 2
**Trafficking**	
ARL8A	ADP-ribosylation factor-like 8A
CALR	Calreticulin
DNAJC13	DnaJ heat shock protein family (Hsp40) member C13
DNAJC5	DnaJ heat shock protein family (Hsp40) member C5
FKBP8	FK506 binding protein 8
LNP	Nucleolar and spindle associated protein 1
PRAF2	PRA1 domain family 2
REEP2	Receptor accessory protein 2
REEP5	Receptor accessory protein 5
RTN1	Reticulon 1
RTN3	Reticulon 3
SACM1L	SAC1 suppressor of actin mutations 1-like (yeast)
SCAMP1	Secretory carrier membrane protein 1
SCAMP2	Secretory carrier membrane protein 2
SCAMP3	Secretory carrier membrane protein 3
SEC22B	SEC22 homolog B, vesicle trafficking protein
VAPB	Vesicle-associated membrane protein, associated protein B and C (ALS8)
**Transmembrane**	
ARL6IP5	ADP-ribosylation factor-like 6 interacting protein 5
DDOST	Dolichyl-di-phosphooligosaccharide-protein glycotransferase
GPM6A	Glycoprotein m6a
MAL2	mal, T cell differentiation protein 2
PLLP	Plasma membrane proteolipid
RTN4	Reticulon 4
SV2A	Synaptic vesicle glycoprotein 2 a
SV2B	Synaptic vesicle glycoprotein 2 b
SYNGR1	Synaptogyrin 1
SYNGR3	Synaptogyrin 3
SYP1	Synaptophysin

## Discussion

### AMPA-Containing Vesicles Can Be Specifically Purified From Whole Mouse Brains

Due to their relatively low abundance at synapses compared to other synaptic content (e.g., synaptic vesicles), ATVs have been difficult to characterize in the past. Here, we developed a protocol to specifically purify and enrich ACVs from synaptosome lysate *via* immunoisolation using a monoclonal anti-GluA1 antibody. A key methodological advance compared to previous isolation protocols consists of specific, competitive, elution off the paramagnetic beads using a molar excess of a small peptide that corresponds to the epitope of the GluA1 monoclonal antibody (Figure 1A). While previous approaches more typically used harsh elution conditions to shear or denature all bound components from the beads, our specific and gentle elution method with the GluA1 epitope peptide minimizes contamination by non-specifically bead-bound components. Indeed, when applied to GluA1 KO mice, our immunoisolation method yielded substantially less vesicular material (Figures 1C,E and Supplementary Figures 2, 3), despite the same amount of LP2 input, providing further evidence that immunoisolation *via* peptide elution is specific. Such little material was generated from immunoisolation from GluA1 KO mice LP2 that LC–MS/MS experiments would require impractically large amounts of starting material. Western blot analysis of samples taken from steps in the purification process further supports the specificity of this isolation. Seven cellular components were probed by western blot (Figure 2A). GluA1, the AMPAR subunit being enriched, was present in each step of the purification process and was enriched in the final elution. In contrast, GluN1 (NMDA receptor subunit), PSD-95 (postsynaptic density component), LAMP1 (late endosome component), and golgin (Golgi marker) were all present throughout the purification process but did not bind to beads nor appear in the final eluate. Typically, synaptosomes generated *via* differential centrifugation have primarily been used to study presynaptic components. Our results suggest that synaptosomes present in the crude synaptosome fraction (P3) also preserve postsynaptic components (GluA1, GluN1, and PSD-95) and that these postsynaptic components are also present in LP2, the input fraction for immunoprecipitation and GluA1 peptide elution. Therefore, the described ultracentrifugation protocol generated a fraction containing relevant postsynaptic components. In the subsequent elution step with GluA1 peptide, the postsynaptic GluN1 and PSD-95 components were removed, resulting in GluA1 components that should include ATVs. Although we cannot rule out that some postsynaptic plasma membrane components are present in our isolation, the absence of these other postsynaptic residents strongly argues for the specificity and purity of our sample. Likewise, we cannot rule out that a fraction of the isolated ACVs are pre-synaptic components. However, our synaptosome preparation likely preserves also some postsynaptic ACVs, considering that LTP can be induced in synaptosomes (Corera et al., 2009) and that AMPAR subunits are synthesized in isolated synaptosomes (Maghsoodi et al., 2008). Thus, at least some of the isolated ACVs should be ATVs.

Immunoelectron microscopy analysis further confirmed the specificity of ACV purification. Unsurprisingly, Syb2, a SNARE protein essential for AMPAR insertion during LTP, labeled 82.1% of ACVs (Jurado et al., 2013). In addition, 42.6% of ACVs were positive for the GluA2 subunit of the AMPAR. This aligns well with evidence that GluA1/GluA2 heteromers are the most common AMPAR composition (Lu et al., 2009; Zhao et al., 2019). Furthermore, 36.7% of ACVs were positive for the GluA3 subunit. This could perhaps be reflective of GluA1/A3 heteromers; it has been previously observed that ∼10% of GluA3-containing AMPARs also contain GluA1 (Wenthold et al., 1996; Diering and Huganir, 2018). Alternatively, multiple AMPARs could be contained in the same ACV, and this observation could be reflective of GluA2/A3 heteromers.

Substantial contamination from synaptic vesicles in our preparation is unlikely for several reasons. First, VGLUT1 was not present in the final eluate, as measured by western blot (Figure 1B) and only a small fraction of purified vesicles was positive for VGLUT1 *via* immunoelectron microscopy (Figure 2G). Second, the purified vesicle population with diameters 102.7 ± 50.8 nm is distinct from a typical synaptic vesicle preparation with tightly defined diameters in the range 40–45 nm. Only 3.7% of the purified vesicles had a diameter less than 50 nm (Figure 1D). Third, the purified vesicles were positive for several markers in immunoelectron microscopy that are unlikely to be in synaptic vesicles, including TfR, GluA2, and GluA3 (Figure 2G). Furthermore, immunoisolation from GluA1 knockout mice yielded negligible material. The presence of synaptophysin, typically thought of as a synaptic vesicle maker, on 90.2% of vesicles isolated from wild-type mice is more likely suggestive of the presence of synaptophysin on ATVs as opposed to contamination due to synaptic vesicles. Therefore, our ACV preparation is relatively pure and contains key proteins associated with AMPAR delivery.

Liquid chromatography–tandem mass spectrometry also provided supportive evidence that ACV purification is specific. The GluA1, GluA2, and GluA4 subunits were all identified in the top mass spectrometry hits. GluA3 was also identified but had lower sequence coverage, possibly due to sequence similarity between it and other AMPAR subunits. Several of the top hits identified *via* mass spectrometry were Rab proteins, including Rab5, Rab8, Rab11, and Rab39, all of which are required for AMPAR trafficking (Gerges et al., 2004), and Rab5 (Brown et al., 2005; Hoogenraad et al., 2010), Rab8 (Gerges et al., 2004), and Rab11 (Hoogenraad et al., 2010) all of which localize in the postsynaptic terminal. Rab39 contributes to AMPAR trafficking from the endoplasmic reticulum to the Golgi, and mutations in this protein have been connected to autism spectrum disorders (Mignogna et al., 2015). Rab5 is required for AMPAR endocytosis (Brown et al., 2005), while Rab8 and Rab11 (Brown et al., 2007) are likely involved in AMPAR insertion into the plasma membrane. Mass spectrometry also identified several other proteins associated with AMPAR trafficking, including Lrp1 (Gan et al., 2014), TfR (Liu et al., 2016), Dnajc13 (Perrett et al., 2015), TDP-43 (Schwenk et al., 2016), and ABHD6 (Wei et al., 2016). Furthermore, Lrp1 (Gan et al., 2014) and TfR (Liu et al., 2016) colocalize with AMPARs. Additionally, NSF, AP-2, POR, ABHD6, and SACM1L have direct interactors of AMPARs (Schwenk et al., 2012; Shanks et al., 2012).

### Composition of AMPA-Containing Vesicles

Immunoelectron microscopy combined with vesicle diameter analysis identified at least two possible unique populations of ACVs. Specifically, the cumulative frequency distribution of the average diameters of ACVs labeled with TfR was significantly shifted to larger diameters compared to an independent overall population of ACVs, while the cumulative frequency distribution of the average diameters of ACVs labeled with Syb2 was significantly shifted to smaller diameters (Figure 2I). These larger ACVs were also more likely to contain GluA2 and GluA3. Thus, our GluA1 immunoisolation whole mouse brain isolates at least two populations of vesicles (Figures 2H,I). The smaller-diameter population of vesicles likely represents a fusion-capable population of vesicles due to the prevalence of Syb2, while Syb2 was rarely observed associated with large ACVs. The fusion of Syb2-positive, GluA1-positive ATVs may play a role in LTP (Jurado et al., 2013). Therefore, the small-diameter Syb2-positive, GluA1-positive population of vesicles that we observe may represent ATVs essential for LTP. The larger ACVs containing TfR and a mixed population of AMPAR subunits may represent recycling endosomes.

Liquid chromatography–tandem mass spectrometry of GluA1 immunoisolated samples identified many of the SNARE and SNARE effector proteins involved in AMPAR insertion during LTP (Figure 4 and Table 1), including Stx-3, SNAP-47, and Syb2 (Jurado et al., 2013). Additionally, the N-terminal, Sec1/Munc18-like-binding portion of Stx-3 is essential for LTP (Jurado et al., 2013), providing evidence for the possible role of Munc18 in AMPAR insertion. Munc18-1 is associated with ACVs as observed by mass spectrometry, and combined with evidence that Munc18-1 binds to Stx-3 (Hata and Südhof, 1995), Munc18-1 is a likely candidate for a regulator of AMPAR insertion. In synaptic vesicle fusion, Munc18 stabilizes syntaxin-1A (Südhof, 2013), and Munc13 is required to aid in the transition of the syntaxin/Munc18 complex into the ternary trans-SNARE complex, a critical step to ensure parallel assembly of all SNARE complex components (Ma et al., 2013; Lai et al., 2017; Brunger et al., 2019). After fusion, the ternary SNARE complex is disassembled with the ATPase, NSF, and adaptor protein, SNAP, for use in future fusion events (Söllner et al., 1993; Mayer et al., 1996; Hanson et al., 1997). Therefore, Munc18, Munc13, NSF, and SNAP could also play roles in regulating SNARE assembly and disassembly during ATV fusion. Additionally, LC-MS/MS identified synaptotagmin-2 (Syt2), a calcium sensor that performs equivalent functions to Syt1 (Pang et al., 2006). Only 44.0% of ACVs contained Syt1 (Figure 2G), consistent with the implication of alternative calcium sensors such as Syt2 or Syt7 for AMPAR insertion (Wu et al., 2017). Furthermore, it is worth noting that the exact location of AMPAR insertion is an active area of exploration (Choquet and Hosy, 2020).

### Potential New AMPAR Trafficking Candidates

Liquid chromatography–tandem mass spectrometry and immunoelectron microscopy of GluA1 immunoisolated ACVs revealed several potential new candidates with connections to AMPAR trafficking and neurological disease (Figures 2, 3 and Table 1). Syp1, best known as a synaptic vesicle marker, densely labeled ACVs, and Syp1, Syngr1, and Syngr3 were identified among the top mass spectrometry hits. Previously, synaptophysin and synaptogyrin have been shown to cooperatively contribute to LTP (Janz et al., 1999). Furthermore, Syngr3 may play a role in tauopathies, and the reduction of Syngr3 expression in neurons rescues synaptic plasticity deficits induced by tau (Largo-Barrientos et al., 2021). While important roles for synaptophysin and synaptogyrin have already been confirmed in the presynaptic terminal, the potential for a postsynaptic contribution has yet to be explored. We validated the presence of Syp1 on ACVs (Figures 2C,G), but further studies are needed to quantify the frequency of Syngr1 and Syngr3 on ACVs.

### Connections to Disease

Many of the candidates identified LC-MS/MS of GluA1 immunoisolated ACVs have been implicated in neurological disorders. The knockdown of TDP-43, a protein implicated in amyotrophic laterals sclerosis (ALS) (Sreedharan et al., 2008), decreases the number and motility of Rab-11 endosomes which in turn impairs AMPAR recycling (Esteves da Silva et al., 2015; Schwenk et al., 2016). Furthermore, mutations in VAPB (ALS8) are causative of familial ALS (Chen et al., 2010). LRP1, previously implicated in both Alzheimer’s disease and GluA1 trafficking, was also identified by mass spectrometry (Liu et al., 2010; Gan et al., 2014). LRP1 directly interacts with GluA1 to control its surface expression (Gan et al., 2014). Finally, Dnajc13, a known contributor to Parkinson’s disease (Vilariño-Güell et al., 2014), is involved in endocytosis of AMPARs (Perrett et al., 2015). In sum, these data reinforce AMPAR endocytosis and recycling pathways as pathways that when dysfunctional, contribute directly to neurological disorders.

The molecular characterization of ACVs presented here is the first time ACVs have been isolated and characterized. Our findings are a potential steppingstone in the understanding of molecular interactors for AMPARs and establish a framework for future AMPAR studies.

## Data Availability Statement

The datasets presented in this study can be found in online repositories as source data. The names of the repository/repositories and accession number(s) can be found below: https://datadryad.org/stash, https://doi.org/10.5061/dryad.r2280gbdh, https://doi.org/10.5061/dryad.jdfn2z3bd.

## Ethics Statement

The animal study was reviewed and approved by the Administrative Panel on Laboratory Animal Care (APLAC) at Stanford University (IACUC #29981).

## Author Contributions

JP: conceptualization, data curation, formal analysis, validation, investigation, visualization, methodology, writing – original draft, and writing – review and editing. JL: conceptualization, investigation, methodology, and writing – review and editing. JO-P: formal analysis, validation, investigation, methodology, and writing – review and editing. ALB: supervision. ATB: conceptualization, supervision, funding acquisition, project administration, and writing – review and editing. All authors contributed to the article and approved the submitted version.

## Conflict of Interest

The authors declare that the research was conducted in the absence of any commercial or financial relationships that could be construed as a potential conflict of interest.

## Publisher’s Note

All claims expressed in this article are solely those of the authors and do not necessarily represent those of their affiliated organizations, or those of the publisher, the editors and the reviewers. Any product that may be evaluated in this article, or claim that may be made by its manufacturer, is not guaranteed or endorsed by the publisher.
