# Supplementary material for: Molecular Characterization of AMPA-Receptor-Containing Vesicles
Source: Front Mol Neurosci. 2021 Oct 15;14:754631. doi: 10.3389/fnmol.2021.754631 (PMC8554035; doi:10.3389/fnmol.2021.754631)
Supplement: Supplementary file 1 [file Data_Sheet_1.PDF]

A

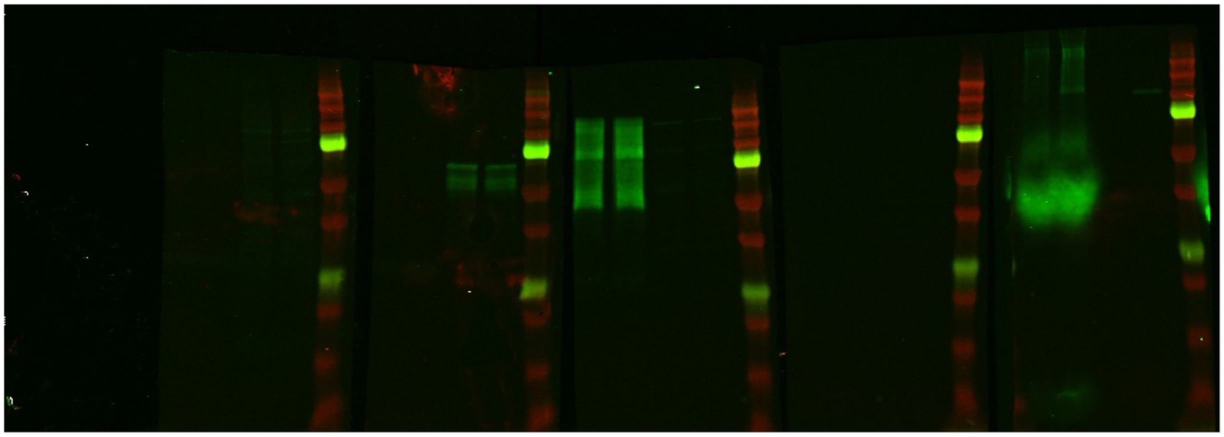

B

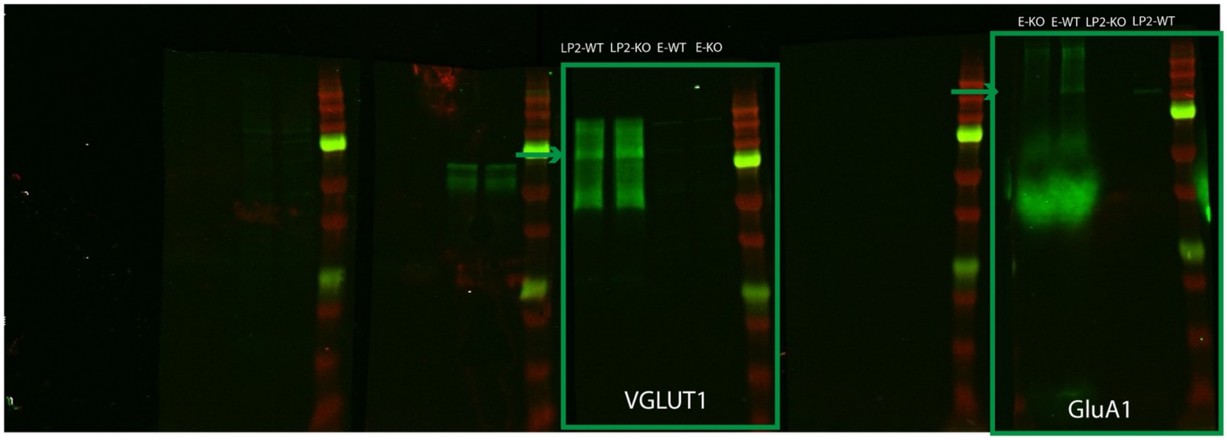

Supplementary Figure 1. Source Data for Figure 1B.
(A) Unaltered Western blots. **(B)** Western blots with lanes labelled and relevant bands labelled (green arrows).
