# Supplementary material for: Molecular Characterization of AMPA-Receptor-Containing Vesicles
Source: Front Mol Neurosci. 2021 Oct 15;14:754631. doi: 10.3389/fnmol.2021.754631 (PMC8554035; doi:10.3389/fnmol.2021.754631)
Supplement: Supplementary file 2 [file Data_Sheet_2.PDF]

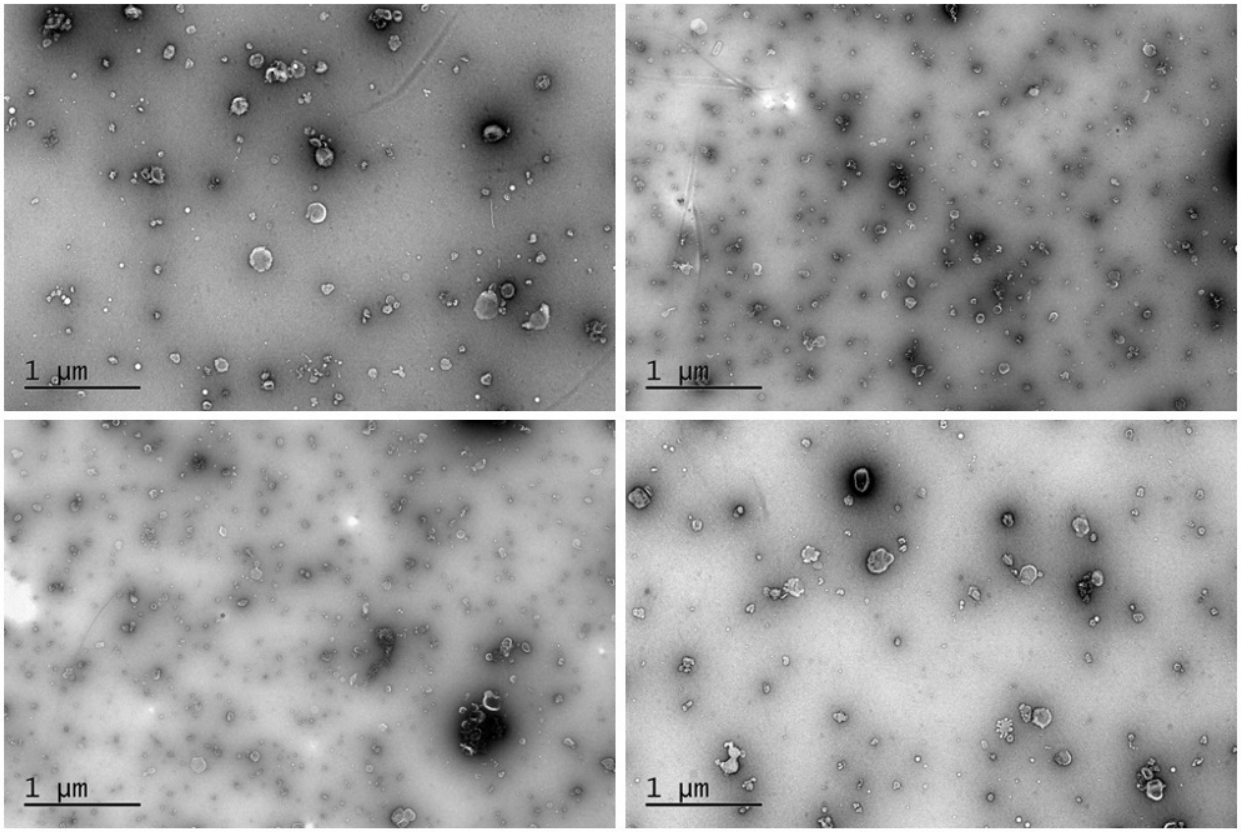

Supplementary Figure 2. Additional examples of negative stain electron microscopy images of GluA1 peptide eluate of wildtype mice (5000x magnification). Additional images are provided in supplementary files.
