# Supplementary material for: Molecular Characterization of AMPA-Receptor-Containing Vesicles
Source: Front Mol Neurosci. 2021 Oct 15;14:754631. doi: 10.3389/fnmol.2021.754631 (PMC8554035; doi:10.3389/fnmol.2021.754631)
Supplement: Supplementary file 3 [file Data_Sheet_3.PDF]

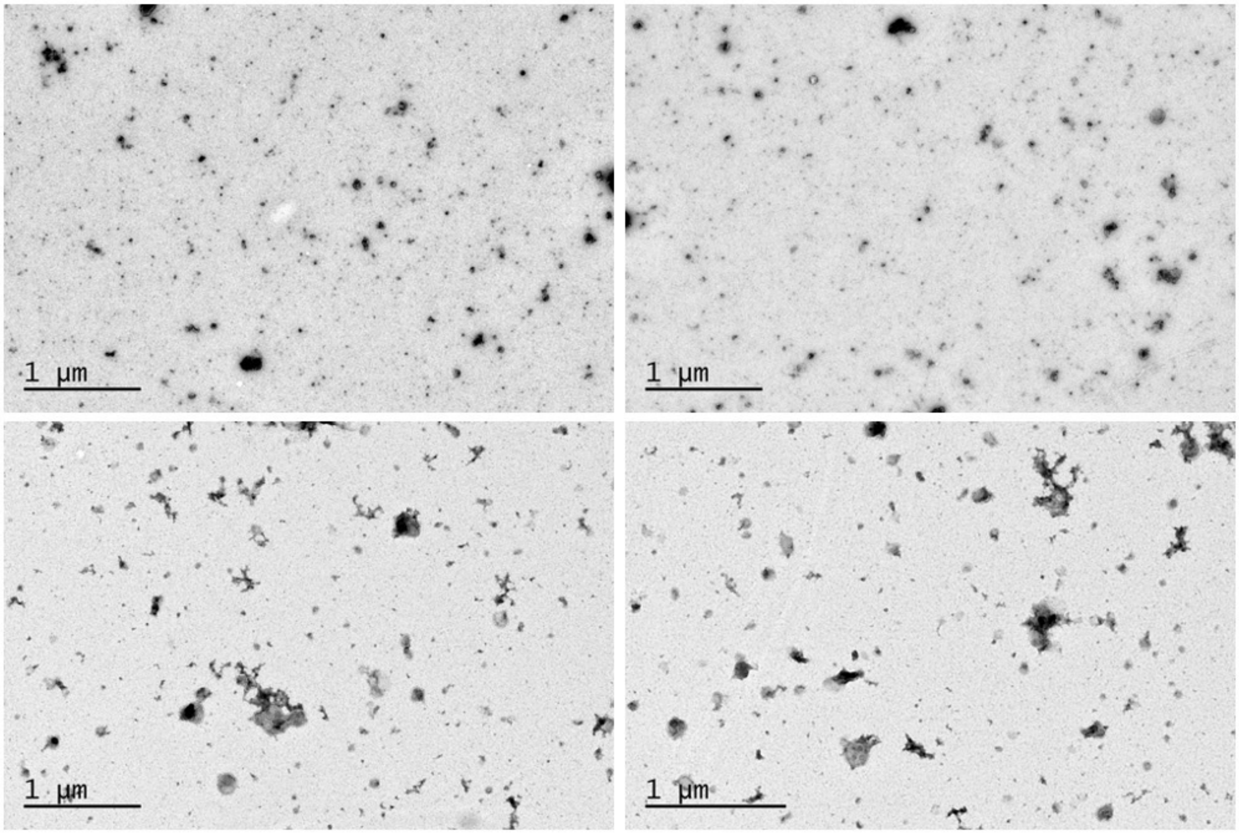

Supplementary Figure 3. Additional examples of negative stain electron microscopy images of GluA1 peptide eluate of GluA1 knockout mice (5000x magnification, except bottom right which is 6000x). Note that the same amount of protein (as assessed by BCA) was inputted into the same immunoisolation step for both wildtype and knockout preparations, making these images comparable to that of the wildtype preparation (Supplementary Figure 2). Additional images are provided in supplementary files (1,500x-12,000 magnification).
