# Supplementary material for: Molecular Characterization of AMPA-Receptor-Containing Vesicles
Source: Front Mol Neurosci. 2021 Oct 15;14:754631. doi: 10.3389/fnmol.2021.754631 (PMC8554035; doi:10.3389/fnmol.2021.754631)
Supplement: Supplementary file 4 [file Data_Sheet_4.PDF]

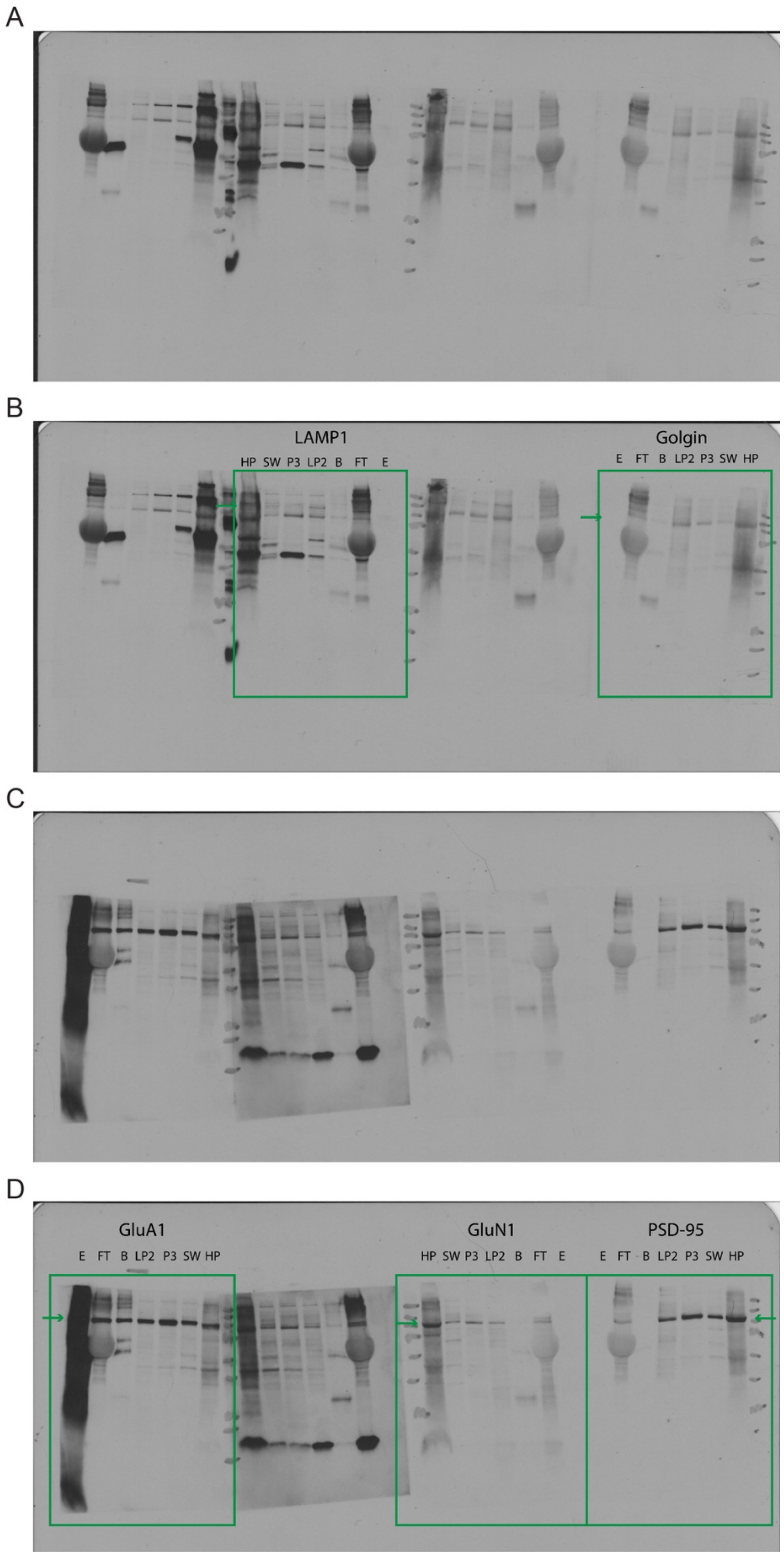

Supplementary Figure 4. Source Data for Figure 1G.
(A & C) Unaltered Western blots. **(B & D)** Western blots with lanes labelled and relevant bands labelled (green arrows).
